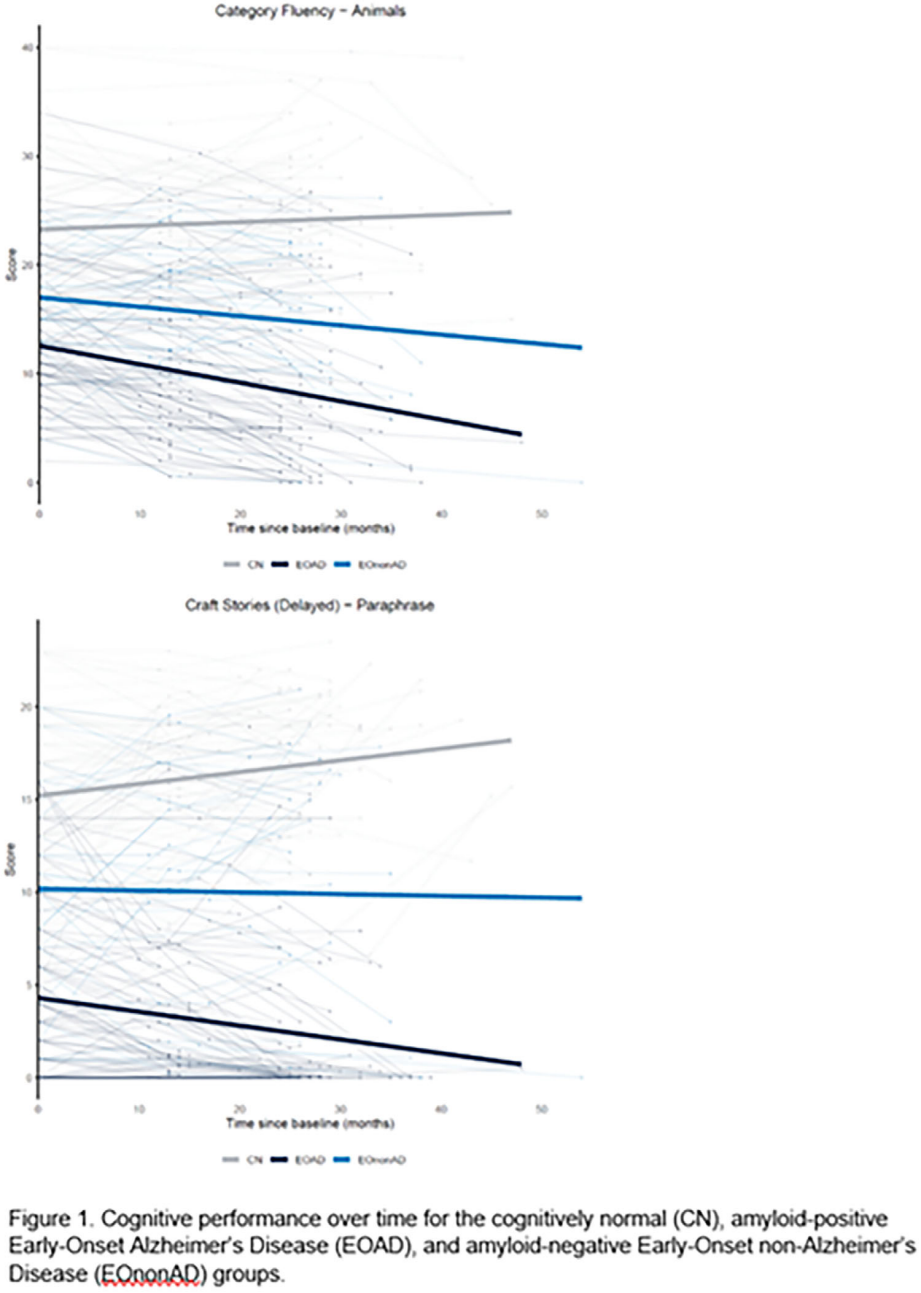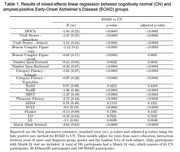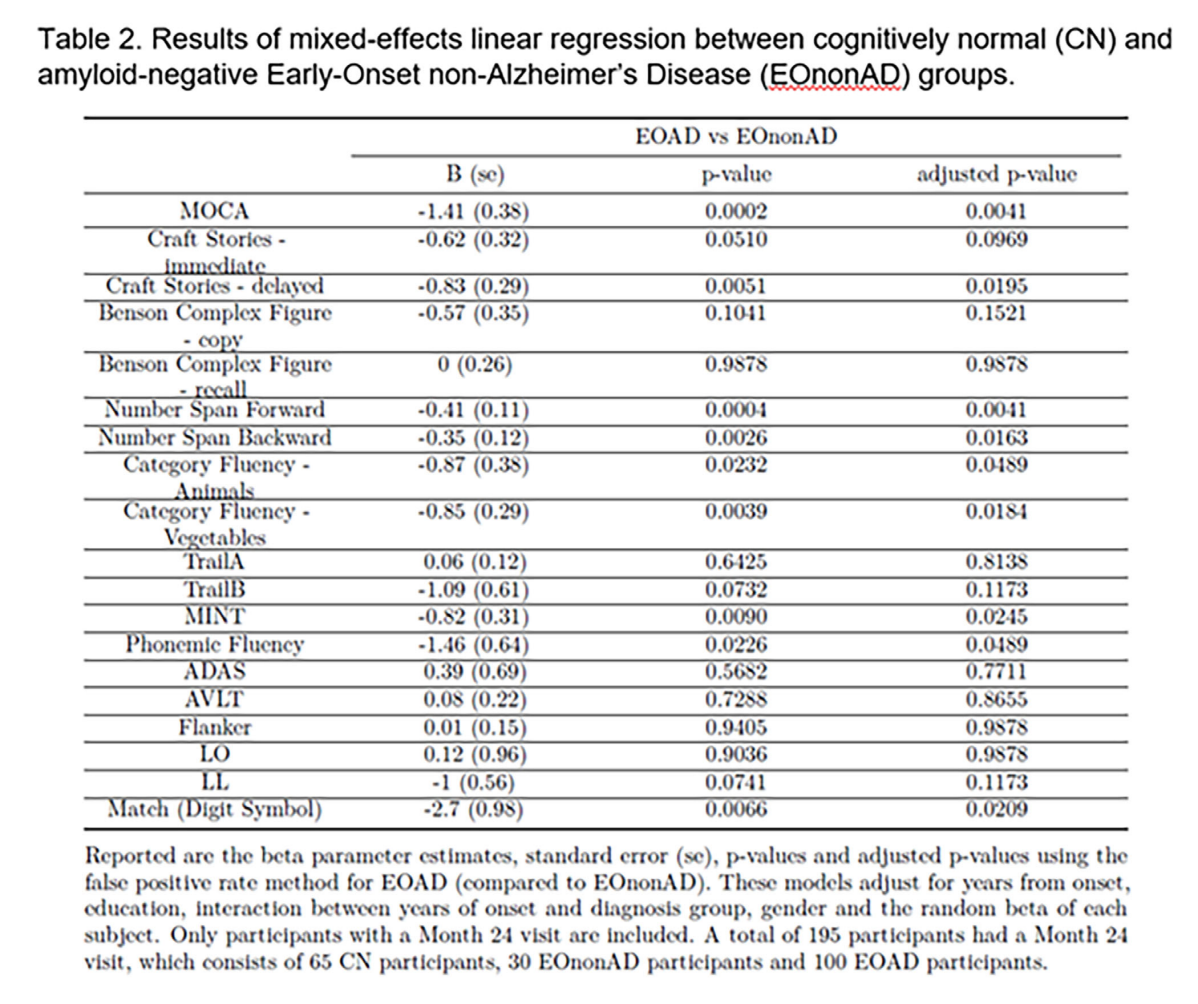# Longitudinal cognitive trajectories in sporadic early‐onset Alzheimer’s Disease: Findings from LEADS

**DOI:** 10.1002/alz.092326

**Published:** 2025-01-03

**Authors:** Dustin B. Hammers, Ani Eloyan, Maryanne Thangarajah, Alexander Taurone, Bonnie Wong, Jeffrey L. Dage, Kelly N. Nudelman, Maria C. Carrillo, Gil D. Rabinovici, Bradford C. Dickerson, Liana G. Apostolova, and ‐ LEADS Consortium

**Affiliations:** ^1^ Indiana University School of Medicine, Indianapolis, IN USA; ^2^ Department of Biostatistics, Brown University, Providence, RI USA; ^3^ Brown University, Providence, RI USA; ^4^ Frontotemporal Disorders Unit, Massachusetts General Hospital, Boston, MA USA; ^5^ Harvard Medical School, Boston, MA USA; ^6^ Department of Neurology, Indiana School of Medicine, Indianapolis, IN USA; ^7^ Department of Medical and Molecular Genetics, Indiana University School of Medicine, Indianapolis, IN USA; ^8^ Alzheimer’s Association, Chicago, IL USA; ^9^ Memory and Aging Center, Weill Institute for Neurosciences, University of California, San Francisco, San Francisco, CA USA; ^10^ Department of Neurology, Harvard Medical School, Boston, MA USA; ^11^ Department of Radiology and Imaging Sciences, Indiana University School of Medicine, Indianapolis, IN USA; ^12^ Department of Neurology, Indiana University School of Medicine, Indianapolis, IN USA

## Abstract

**Background:**

Early Onset Alzheimer’s Disease (EOAD) is a rare condition that manifests prior to the age of 65, and affects approximately 5% of patients with Alzheimer’s disease. The Longitudinal Early‐Onset Alzheimer’s Disease Study (LEADS) is the largest prospectively‐evaluated cohort of participants with sporadic EOAD in the United States, initiated to better understand the features of this condition. The current analyses sought to examine longitudinal cognitive trajectories of patients with EOAD over time.

**Method:**

Data from 100 participants with amyloid‐positive EOAD, 30 participants with amyloid‐negative cognitive impairment (EOnonAD), and 65 cognitively normal age‐matched participants were compared. All had at least three study visits. Cognitive trajectories across a comprehensive cognitive battery across 24‐56 months were examined using mixed‐effects modeling, including the years of onset x diagnostic group interaction controlling for years since onset, education, sex, and the random effect of each participant.

**Result:**

Across all measures, clinical groups generally displayed declines over time, with performances for the EOAD group tending to approach the lower limit of performance ranges (**Figure 1**). Relatedly, significantly greater slopes of decline were seen over time for the EOAD group than the CN group across all cognitive domains evaluated (ps<.001; **Table 1**). When comparing between clinical groups, greater declines were also evident for the EOAD group relative to the EOnonAD group for a screener of global cognition, and for specific measures of attention, verbal fluency, processing speed, language, and delayed story recall (ps .001 to .049; **Table 2**). No differences in trajectory were observed between clinical groups for unstructured verbal memory, visual memory, or visuospatial skills (ps>.05; **Tables 1 and 2**).

**Conclusion:**

In addition to worse cognition at baseline, sporadic EOAD participants displayed pronounced declines in cognition over 24‐56 months across all domains evaluated. Relative to the EOnonAD group, cognitive trajectories appear to be worse predominantly for executive and attentional processes, with variability across episodic memory tasks. This suggests that EOAD pathology is not solely directed at memory functioning. Future research will focus on comparing cognitive trajectories of EOAD and late‐onset AD, in an effort to understand similarities and differences in the types and rates of cognitive trajectories.